# Application of MALDI-TOF MS to rapid identification of anaerobic bacteria

**DOI:** 10.1186/s12879-019-4584-0

**Published:** 2019-11-07

**Authors:** Ying Li, Mingzhu Shan, Zuobin Zhu, Xuhua Mao, Mingju Yan, Ying Chen, Qiuju Zhu, Hongchun Li, Bing Gu

**Affiliations:** 1 School of Medical Technology, Xuzhou Medical University, Xuzhou, 221004 China; 2 Department of Genetics, Xuzhou Medical University, Xuzhou, 221004 China; 3 Department of Clinical Laboratory, Yixing People’s Hospital, Wuxi, 214200 China; 4 Jiangsu Key Laboratory of Brain Disease Bioinformation, Xuzhou Medical University, Xuzhou, 221004 China; 5 Department of Laboratory Medicine, Affiliated Hospital of Xuzhou Medical University, Xuzhou, 221002 China

**Keywords:** MALDI-TOF MS, Anaerobes, Bacteria identification

## Abstract

**Background:**

Matrix-assisted laser desorption ionization-time of flight mass spectrometry (MALDI-TOF MS) has been rapidly developed and widely used as an analytical technique in clinical laboratories with high accuracy in microorganism identification.

**Objective:**

To validate the efficacy of MALDI-TOF MS in identification of clinical pathogenic anaerobes.

**Methods:**

Twenty-eight studies covering 6685 strains of anaerobic bacteria were included in this meta-analysis. Fixed-effects models based on the *P*-value and the I-squared were used for meta-analysis to consider the possibility of heterogeneity between studies. Statistical analyses were performed by using STATA 12.0.

**Results:**

The identification accuracy of MALDI-TOF MS was 84% for species (I^2^ = 98.0%, *P* < 0.1), and 92% for genus (I^2^ = 96.6%, *P* < 0.1). Thereinto, the identification accuracy of *Bacteroides* was the highest at 96% with a 95% CI of 95–97%, followed by *Lactobacillus* spp., *Parabacteroides* spp., *Clostridium* spp., *Propionibacterium* spp., *Prevotella* spp., *Veillonella* spp. and *Peptostreptococcus* spp., and their correct identification rates were all above 90%, while the accuracy of rare anaerobic bacteria was relatively low. Meanwhile, the overall capabilities of two MALDI-TOF MS systems were different. The identification accuracy rate was 90% for VITEK MS vs. 86% for MALDI biotyper system.

**Conclusions:**

Our research showed that MALDI-TOF-MS was satisfactory in genus identification of clinical pathogenic anaerobic bacteria. However, this method still suffers from different drawbacks in precise identification of rare anaerobe and species levels of common anaerobic bacteria.

## Background

Anaerobic bacteria exist as part of the normal flora in the human intestinal tract, oral cavity and urogenital tract **[**1], and can cause infectious diseases as a result of impairment to the microenvironment and/or immune system. Anaerobic infection can also be induced by deep wounds accompanied with facultative anaerobes and aerobic bacteria invasion. Invasive anaerobic infections are life threatening, and the mortality rate of anaerobic bacteremia is high as 40% [2]. Thus, the accurate and fast identification of anaerobic bacteria is pivotal to prompt antimicrobial treatments. Conventional anaerobe identification methods are cumbersome, time-consuming, and costly. It requires a long-term cultivation (not less than 24 h) to obtain enough inocula. In addition, the identification work is complex, including colony traits, colony morphology, and staining results. Meanwhile, it is difficult to identify rare or newly identified species by using conventional phenotyping methods and commercial kits [3]. Real-time, fast, high-throughput, high-sensitivity, high-selectivity, and low cost have been the goals pursued by analysts in modern analytical science.

The modern mass spectrometry technology enhances the understanding about the whole biological system through direct analysis of biological molecules such as proteins, lipids, carbohydrates and amino acids [4], which has been applied to the field of life science [5]. As an emerging technology, matrix-assisted laser desorption ionization-time of flight mass spectrometry (MALDI-TOF MS) has been widely used in clinical microbial diagnosis in the past decade. It is gradually replacing the traditional identification methods [6, 7]. MALDI-TOF MS is a rapid mass spectrometry technology developed in the late 1980s with relatively high sensitivity to various samples types. It is a useful, fast and accurate tool for routine laboratory analysis and has been used to identify mycobacteria [8, 9], nocardia [10], yeasts [11, 12] and anaerobes [13, 14] isolated from solid media of clinical specimens. At present, there are few studies to evaluate the efficacy of identifying anaerobic bacteria by MALDI-TOF MS. The aim of the present meta-analysis is to determine the reliability and effectiveness of mass spectrometry as a routine diagnostic method for anaerobic bacteria by searching related publications in the literature.

## Methods

### Search strategy

The scientific literature was extensively searched using the MeSH terms “maldi-ms” and “anaerobic bacteria” to query the electronic database of Medline and Web of science (up to 1 April 2018). Selected articles contained studies involving the identification of anaerobes by MALDI-TOF MS. The references cited in these articles were examined to determine other articles. The meta-analysis was performed by referring to (when appropriate) the PRISMA guidelines [15]. EndNote X8 (Thomson Reuters) was used for literature management. We read the titles and abstracts of each searched publication and selected only those relevant articles for full-text reading. There are no restrictions on the language, publication status and geographical distribution of publications.

### Inclusion and exclusion criteria

We set up the criteria for the inclusion and exclusion of the literature. The inclusion criteria were as follows: (1) the study objective: the clinical specimens were identified as anaerobic bacteria by reference methods (16S rRNA gene sequencing); (2) the study method: the identification of anaerobes by MALDI-TOF MS; (3) the research objective: the accuracy of MALDI-TOF MS identification of anaerobes. The exclusion criteria included the following aspects: review articles, reviews, case reports, scientific abstracts and lectures; common anaerobes with fewer than 10 strains of anaerobes and less than 5 uncommon anaerobes; direct identification of bacteria in the positive blood culture bottle; The target bacteria could not be extracted, and pathogenic microorganisms or industrial environmental microbes of plants or animals were identified.

The identification criteria of MALDI-TOF MS in the included studies were as follows: a score of ≥2.0 was considered an accurate species-level identification; ≥ 1.7 but < 2.0, a probable genus-level identification; an isolate with a score < 1.7 was considered as “unidentified”; and an isolate identified as another species or genus was considered to be “misidentification”.

### Quality assessment

What is important in meta-analysis is whether heterogeneity exists in the included studies and the possible reasons for the existence of heterogeneity, because heterogeneity may lead to deviations in meta-analysis results - known as “mixed apples and oranges” [16]. The sources of heterogeneity can be divided into, different research designs, different experimental conditions, different definitions of exposure and/or outcomes, different measurement methods, and the existence of other interference factors, i.e. covariates. In addition, low-quality literature will bring significant heterogeneity [17]. The following modified criteria, referring to the quality assessment for studies of diagnostic accuracy (QUADAS) [18], were used to assess the quality of original studies: study design, category and geographical distribution of strains, blinded status, reference methods, threshold, strain source, and system database.

### Assessment of publication bias and influence analysis

According to statistics, the studies of positive results are more likely to publish than those of negative results, but it could not really represent the overall study population. In fact, the samples may be less representative. This result is called “publication bias” in statistics [17]. Funnel diagrams are commonly used graphical tests to assess publication bias in meta-analysis [19]. Egger’ s linear regression test of funnel plot asymmetry at the genus level and Begg’s rank correlation (with continuity correction) showed that little publication bias was detected in this review (t = − 1.54 and *P* = 0.123 for Egger’ s Test; z = − 0.35 and *P* = 0.727 for Begg’s Test).

## Results

### Results of the systematic literature search

**Fig. 1:**
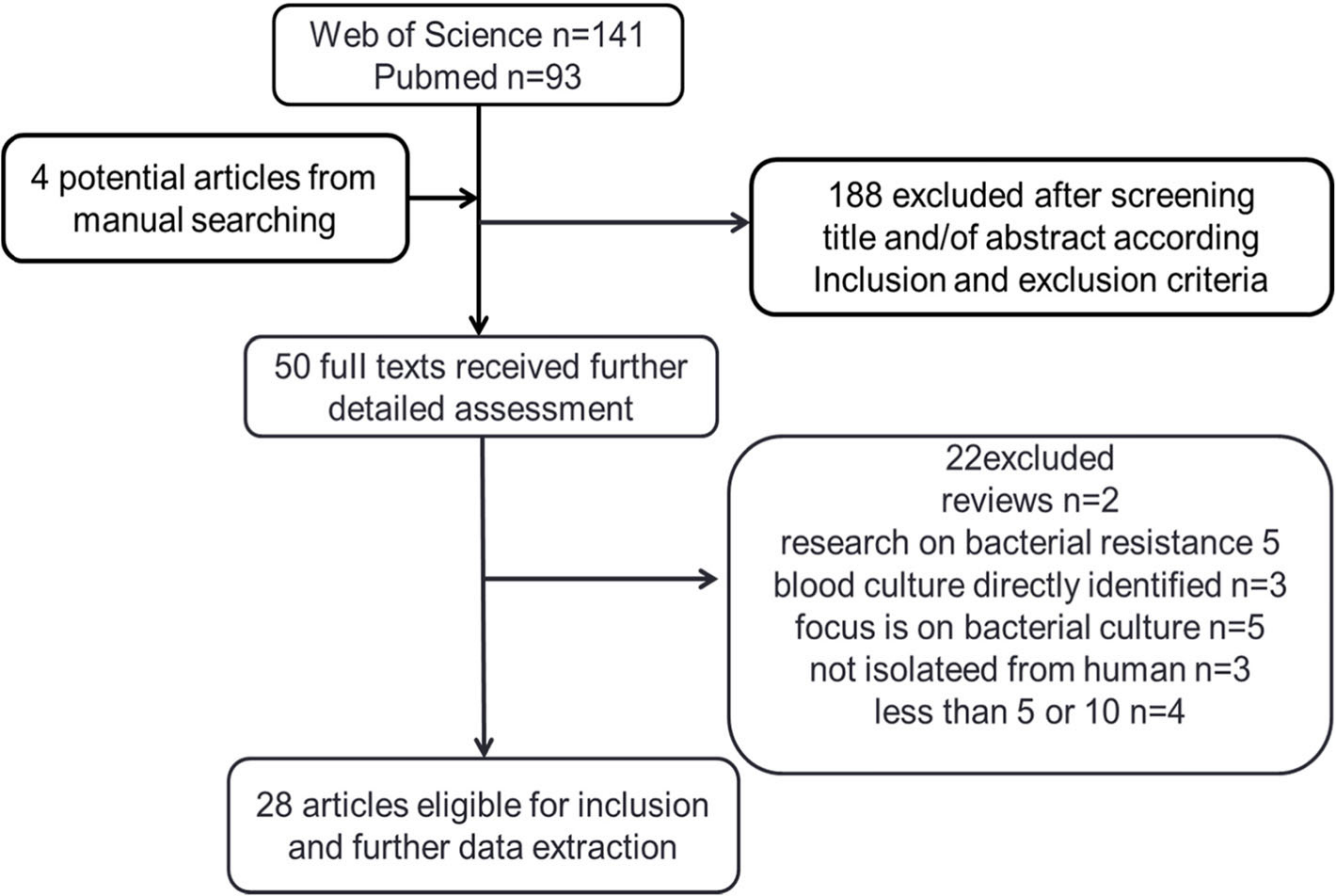
Flow diagram for selection of studies

A total of 234 articles were retrieved from the electronic database. Additional four articles were identified through manual search, bibliographic search, and commentator suggestions. Finally, 28 studies were included according to the defined inclusion and exclusion criteria (Fig. 1). Countries and study periods included in all articles were shown in Table 1. The geographical distributions of the literature were Asia (5, 17.86%), Australia (1, 3.57%), South America (1, 3.57%), North America (4, 14.29%) and Europe (17, 60.71%), containing 24 cities in 14 countries.

**Table 1:** Geographical distributions and study periods of all included studies

Author (publication year)	Country	City	Period of the study
Lucia Sanchez Ramos (2018) [20]	Germany	Leipzig	NM
Mervi Gürsoy (2017) [21]	Finland	Turku	NM
Belén Rodríguez-Sánchez (2017) [22]	Spain	Madrid	January 2010 to August 2012.
A.C.M. Veloo (2016) [23]	The Netherlands	Groningen	NM
Tomoyuki Yunoki (2016) [24]	Japan	Kyoto	June 2013 to May 2014
Sung Jin Jo, M.D. (2015) [25]	Korea	Seoul	January to February 2015
NINA HANDAL (2014) [26]	Norway	Lørenskog	January 2009 to December 2013
Wonmok Lee, M.D. (2014) [27]	Korea	Seoul	2011
M.J. Barba (2014) [28]	Spain	A Coruña	2007–2014
Roy Chean (2014) [29]	Australia	Melbourne	2000–2010
Mariela S. Záratea (2014) [30]	Argentina	Ciudad Autónoma de Buenos Aires	NM
Yang Li (2014) [31]	China	Nanjing	NM
Yen-Michael S. Hsu (2014) [32]	USA	St. Louis	NM
Susanna K P Lau (2013) [33]	China	Hong Kong	NM
O. Garner (2013) [34]	USA	St. Louis	January 2012 to August 2012.
Melody Barreau(2013) [35]	France	Marseille	2010–2013
L. Coltella (2013) [7]	Italy	Rome	June 2010 to October 2011
Bryan H. Schmitt (2012) [36]	USA	Minnesota	2012
N. Wüppenhorst (2012) [37]	Germany	Freiburg	NM
Silvia Vega-Casta˜no (2012) [38]	Spain	Salamanca	NM
Rémi Fournier (2012) [39]	France	Lille	NM
M. Knoester (2012) [40]	The Netherlands	Leiden	January 2010 to February 2011
D. P. Fedorko (2012) [41]	USA	Bethesda	NM
Ulrik Stenz Justesen (2011) [14]	Denmark	Vejle	November 2007 to October 2010
Esther Culebras (2011) [42]	Spain	Madrid	2004–2006
Bernard La Scola (2011) [13]	France	Marseille	2009–2010
A. C. M. Veloo (2011) [43]	The Netherlands	Leiden	NM
A.C.M. Velooa (2011) [44]	The Netherlands	Groningen	NM

*NM* Not mentioned in the article

### Bacterial isolates

After comprehensive and detailed data compilation, we collected 6685 (Additional file 1) strains of anaerobic bacteria. The most 4 common genera (> 500) in this article were *Bacteroides* spp. (1952), *Clostridium* spp. (1599), *Propionibacterium* spp. (611) and *Prevotella* spp. (509). A total of 5125 anaerobic bacteria were analyzed by MALDI biotyper, and VITEK MS analyzed a total of 1609 anaerobic bacteria. In addition, 49 anaerobic bacteria were analyzed by both MALDI-TOF MS systems.

### Performance of the MS system

**Fig. 2:**
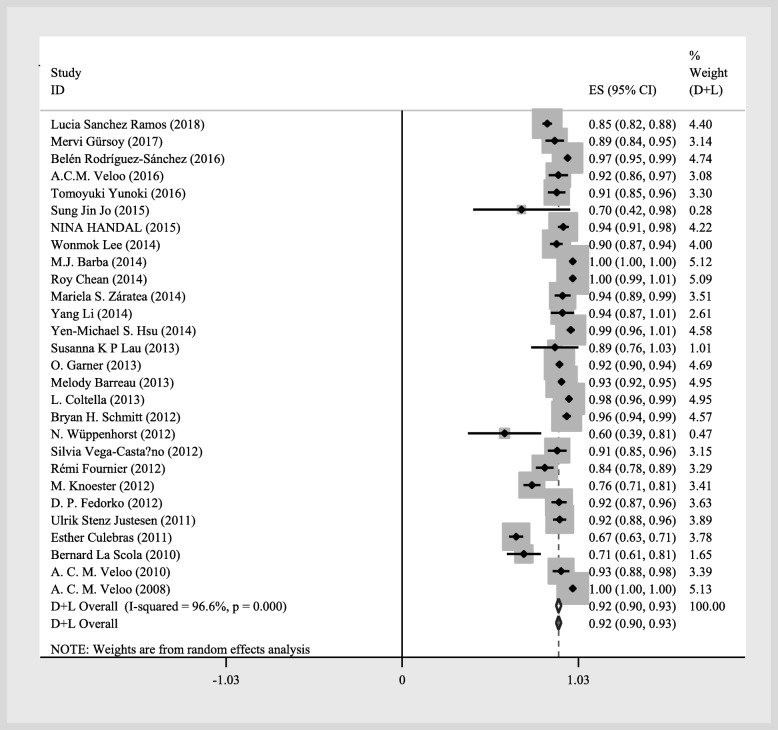
Forest plot for the meta-analysis of the gross identification ratio at the genus level

**Fig. 3:**
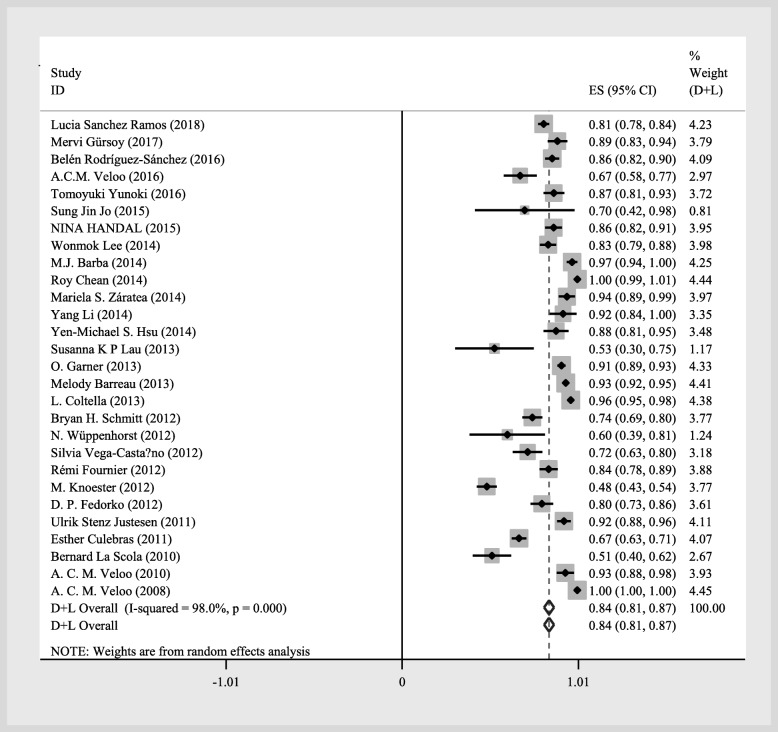
Forest plot for the meta-analysis of the gross identification ratio at the species level

The overall statistical results of the meta-analysis at the genus and species levels identification were summarized using a forest plots of random-effects model (Figs. 2 and 3) [3, 13, 14, 20–44]. Of these, 6008 (92%; 95% CI of 90 to 93%) were correctly identified at the genus level, while 5656 (84%; 95% CI of 81 to 87%) were correctly identified at the species level by MALDI-TOF MS using a random-effects model.

The pooled identification results of MALDI-TOF MS by random-effects for all anaerobic genera were shown in Table 2. The overall correct identification ratio of MALDI-TOF MS to anaerobic bacteria ranged from 60 to 100% at the genus level and ranged from 51 to 100% at the species level. Significant heterogeneity was found both at the genus level (*P* < 0.001; I^2^ = 96.6%) and the species level (*P* < 0.001; I^2^ = 98.0%). Identification accuracy of *Bacteroides* spp. was the highest at 96% with a 95% CI of 95 to 97%. The higher proportion of anaerobic bacteria was *Lactobacillus* spp., *Parabacteroides* spp., *Clostridium* spp., *Propionibacterium* spp., *Prevotella* spp., *Veillonella* spp. and *Peptostreptococcus* spp. The correct identification rate was higher than 90%. Identification accuracy of *Bifidobacterium* spp., *Solobacterium* spp., *Finegoldia* spp., *Capnocytophaga* spp., *Parvimonas* spp., *Peptoniphilus* spp., *Slackia* spp., *Actinomyces* spp., *Ruminococcus* spp. and *Tissierella* spp. was similar with an overall correct identification ratio at 80%, followed by *Fusobacterium* spp., *Eggerthella* spp. with an identification proportion above 70%. Identification accuracy of *Actinobaculum* spp., *Atopobium* spp., *Anaerococcus* spp. and *Flavonifracter* spp. was similar with an overall correct identification ratio at 60%. The lowest performance of MALDI-TOF MS was in *Eubacterium* spp., *Bilophila* spp., *Butyricimonas* spp. and *Porphyromonas* spp. (50%). Multiple factors contributed to this result, including the category of strains, the proportion of common and unusual species, or the reference library version.

**Table 2:** Identification accuracy rate of all anaerobic genera

Genus	Proportion	95%CI	Weight%
*Bacteroides*	96%	95–97%	6.79
*Lactobacillus*	95%	89–102%	5.16
*Parabacteroides*	94%	87–101%	4.88
*Clostridium*	92%	90–93%	6.73
*Propionibacterium*	91%	89–93%	6.55
*Prevotella*	91%	88–93%	6.48
*Veillonella*	91%	85–197%	5.25
*Peptostreptococcus*	90%	85–95%	5.60
*Bifidobacterium*	89%	76–103%	2.60
*Solobacterium*	88%	73–104%	2.27
*Finegoldia*	87%	83–91%	5.96
*Capnocytophaga*	86%	60–112%	1.01
*Parvimonas*	86%	82–91%	5.78
*Peptoniphilus*	86%	81–91%	5.59
*Slackia*	83%	67–98%	2.24
*Actinomyces*	81%	74–89%	4.66
*Ruminococcus*	80%	45–115%	0.59
*Tissierella*	80%	45–115%	0.59
*Fusobacterium*	79%	74–84%	5.61
*Eggerthella*	74%	63–85%	3.39
*Actinobaculum*	68%	46–91%	1.26
*Atopobium*	68%	51–85%	1.92
*Anaerococcus*	64%	54–73%	3.92
*Flavonifracter*	63%	29–96%	0.64
*Eubacterium*	57%	20–94%	0.55
*Bilophila*	56%	23–88%	0.68
*Butyricimonas*	56%	23–88%	0.68
*Porphyromonas*	50%	36–64%	2.59

### Subgroup meta-analyses

We selected the genera (sample number not smaller than 5) identified by MALDI biotyper and VITEK MS to compare the identification accuracy for the same genus of the two systems (Table 3). The identification accuracy rate of MALDI biotyper was higher than VITEK MS for *Parabacteroides* spp., *Eggerthella* spp., *Peptostreptococcus* spp., *Parvimonas* spp., *Bacteroides* spp., *Clostridium* spp. and *Peptoniphilus* spp., and the efficacy of the two systems were similar for *Prevotella* spp. and *Actinomyces* spp. However, the heterogeneity of MALDI biotyper was more significant. In addition, the correct rate of MALDI biotyper for some strains (such as *Finegoldia* spp. and *Fusobacterium* spp.) was lower than VITEK MS, and the heterogeneity of MALDI biotype was higher than the latter. To sum up, the results of Table 3 showed that the correct rate of MALDI biotyper identification of anaerobic bacteria was higher than that of VITEK MS, while the heterogeneity of the MALDI biotyper was more significant.

**Table 3:** Accuracy of MALDI-TOF MS identification

Genus	Number^a^	MALDI biotyper^b^	Vitek^b^
*Parabacteroides*	42/6	100% (I^2^ = 0.0%, *P* > 0.01)	72% (I^2^ = 83.3%, *P* > 0.01)
*Peptostreptococcus*	41/91	100% (I^2^ = 0.0%, *P* > 0.01)	97% (I^2^ = 76.6%, *P* > 0.01)
*Eggerthella*	36/26	100% (I^2^ = 21.8%, *P* > 0.01)	77% (I^2^ = 0.0%, *P* > 0.01)
*Parvimonas*	205/7	100% (I^2^ = 35.5%, *P* > 0.01)	98% (I^2^ = 19.0%, *P* > 0.01)
*Clostridium*	779/820	98% (I^2^ = 63.5%, *P* < 0.01)	94% (I^2^ = 96.7%, *P* < 0.01)
*Finegoldia*	233/17	98% (I^2^ = 77.8%, *P* < 0.01)	99% (I^2^ = 2.0%, *P* > 0.01)
*Prevotella*	404/105	92% (I^2^ = 84.1%, *P* < 0.01)	92% (I^2^ = 0.0%, *P* > 0.01)
*Bacteroides*	1517/435	97% (I^2^ = 89.4%, *P* < 0.01)	96% (I^2^ = 74.8%, *P* < 0.01)
*Fusobacterium*	214/34	91% (I^2^ = 89.7%, *P* < 0.01)	92% (I^2^ = 84.8%, *P* = 0.01)
*Propionibacterium*	605/6	90% (I^2^ = 91.6%, *P* < 0.01)	100% (I^2^ = 0.0%, *P* > 0.01)
*Peptoniphilus*	41/91	85% (I^2^ = 92.4%, *P* < 0.01)	62% (I^2^ = 96.8%, *P* < 0.01)
*Actinomyces*	79/28	74% (I^2^ = 94.5%, *P* < 0.01)	74% (I^2^ = 0.0%, *P* > 0.01)

^a^The left side of / is the number of samples of MALDI biotyper, and the right side of / is the number of samples of Vitek

^b^The higher I-square values combined *P* value more than 0.1 mean the higher heterogeneity between those studies

In additional, the identification rate of anaerobic bacteria in European countries (species: 84%, genus: 88%) was lower than that in Asia (species: 84%, genus: 91%) and North America (species: 86%, genus: 94%). The protocol for the studies at different cities was the same. A total of 21 articles reported on the media, including anaerobic horse blood agar, chocolate agar, blood culture bottle, schaedler agar, bacteroides bile esculin agar, CDC anaerobic blood agar, brucella blood plates, columbia blood plates and blood plates. Among them, brucella and columbia blood plates were two most frequently used media (species 73% and genus 92%; species 73% and genus 75%).

It was worth noting that VITEK MS incorrectly identified *Actinomyces georgiae* as *Capnocytophaga gingivalis*, MALDI biotyper incorrectly identified *Clostridium* spp. as *Enterococcus* spp. (Table 4), and MALDI biotyper also incorrectly identified some rare anaerobic bacteria *Mogibacterium timidum* and *Parvimonas micra* as other bacteria, probably due to the lack of corresponding standard spectra in the database.

**Table 4:** Common misidentification pattern in these studies

Sequence identifcation	MALDI-TOF MS identifcation	System	Reference
*Actinomyces georgiae*	*Capnocytophaga gingivalis*	bioMérieux Vitek MS	[27]
*Actinomyces graevenitzii*	*Actinomyces turicensis*	Bruker MALDI Biotyper	[7]
*Actinomyces meyeri*	*Actinomyces odontolyticus*	Bruker MALDI Biotyper	[58]
*Actinomyces viscosus*	*Actinomyces oris*	Bruker MALDI Biotyper	[58]
*Anaerococcus hydrogenalis*	*Bacteroides fragilis*	Bruker MALDI Biotyper	[24]
*Anaerococcus tetradius*	*Brevibacillus spp.*	bioMérieux Vitek MS	[31]
*Bacteroides cellulosilyticus*	*Bacteroides intestinalis*	Bruker MALDI Biotyper	[3]
*Bacteroides dorei*	*Bacteroides vulgatus*	Bruker MALDI Biotyper	[28, 58]
*Bacteroides faecis*	*Bacteroides thetaiotaomicron*	Bruker MALDI Biotyper	[58]
*Bacteroides faecis*	*Bacteroides thetaiotaomicron*	bioMérieux Vitek MS	[27]
*Bacteroides nordii*	*Bacteroides thetaiotaomicron*	bioMérieux Vitek MS	[27]
*Bacteroides vulgatus*	*Bacteroides eggerthii*	bioMérieux Vitek MS	[31]
*Clostridium baratii*	*Clostridium paraputrificum*	bioMérieux Vitek MS	[20]
*Clostridium beijerinckii*	*Clostridium butyricum*	bioMérieux Vitek MS	[20]
*Clostridium bifermentans*	*Clostridium sordellii*	bioMérieux Vitek MS	[20]
*Clostridium bolteae*	*Clostridium clostridioforme*	bioMérieux Vitek MS	[27]
*Clostridium butyricum*	*Clostridium beijerinckii*	bioMérieux Vitek MS	[20]
*Clostridium cadaveris*	*Clostridium sordellii*	bioMérieux Vitek MS	[20]
*Clostridium clostridioforme*	*Bacillus ssp.*	bioMérieux Vitek MS	[20]
*Clostridium difficile*	*Clostridium septicum*	bioMérieux Vitek MS	[20]
*Clostridium difficile*	*Enterococcus faecium*	Bruker MALDI Biotyper	[24]
*Clostridium histolyticum*	*C. sordellii/C. septicum*	bioMérieux Vitek MS	[20]
*Clostridium limosum*	*Clostridium tyrobutyricum*	bioMérieux Vitek MS	[20]
*Clostridium perfringens*	*Enterococcus faecalis*	Bruker MALDI Biotyper	[24]
*Clostridium sordellii*	*Clostridium bifermentans*	bioMérieux Vitek MS	[20]
*Clostridium sporogenes*	*C.difficile/ C.glycolicum*	bioMérieux Vitek MS	[20]
*Clostridium tetani*	*Clostridium septicum*	bioMérieux Vitek MS	[20]
*Fusobacterium nucleatum*	*Enterococcus faecalis*	Bruker MALDI Biotyper	[24]
*Fusobacterium nucleatum*	*Fusobacterium naviforme*	Bruker MALDI Biotyper	[26]
*Mogibacterium timidum*	*Clostridium halophilum*	Bruker MALDI Biotyper	[58]
*Parvimonas micra*	*Slackia exigua*	Bruker MALDI Biotyper	[24]
*Peptoniphilus Indolicus*	*Peptoniphilus harei*	Bruker MALDI Biotyper	[28, 58]
*Porphyromonas gulae*	*Porphyromonas gingivalis*	Bruker MALDI Biotyper	[58]
*Prevotella bivia*	*Streptococcus anginosus*	Bruker MALDI Biotyper	[24]
*Prevotella denticola*	*Bacteroides fragilis*	Bruker MALDI Biotyper	[24]
*Prevotella oralis*	*Prevotella nanciencis*	Bruker MALDI Biotyper	[58]
*Prevotella oris*	*Prevotella buccae*	Bruker MALDI Biotyper	[24]
*Veillonella dispar*	*Veillonella parvula*	Bruker MALDI Biotyper	[7]
*Veillonella dispar*	*Veillonella parvula*	bioMérieux Vitek MS	[27]

## Discussion

MALDI-TOF MS, based on the microbial identification of characteristic protein fingerprints of bacteria, usually takes only a few minutes to rapidly identify species of different microorganisms, thus greatly shortening the detection time and improving the diagnostic efficiency of infectious diseases. It is usually difficult to isolate and culture anaerobic bacteria by conventional approaches, and MALDI-TOF MS provides a useful technology for their identification. In this study, we conducted a meta-analysis to analyze the differences in independent research results by addressing heterogeneity between studies in an attempt to shed new light on the identification of anaerobic bacteria by MALDI-TOF MS [45, 46].

According to the inclusion and exclusion criteria, 28 anaerobic genera were included and assessed critically using two currently available MALDI-TOF MS systems. It is known that anaerobes are more difficult to be identified in clinical laboratories [47]. However, using MALDI-TOF MS, the overall identification accuracy of anaerobic bacteria was 92% (95% CI of 0.90 to 0.93) at the genus level in 28 included articles with 6685 various anaerobes isolates. These results indicate that MALDI-TOF MS is a qualified method for accurate and rapid identification of pathogenic anaerobes. At the same time, we noticed that the identification property of MALDI-TOF MS against common anaerobe isolate species was variable. Among them, the correct rate was more than 80% for 18 anaerobic genera (*Bacteroides* spp., *Lactobacillus* spp., *Parabacteroides* spp., *Clostridium* spp., ect.), 60–80% for 6 anaerobic genera (*Fusobacterium* spp., *Eggerthella* spp., *Actinobaculum* spp., *Atopobium* spp., *Anaerococcus* spp., *Flavonifracter* spp.,), and lower than 60% for the other 4 anaerobic genera (*Eubacterium* spp., *Bilophila* spp., *Butyricimonas* spp. and *Porphyromonas* spp.). The different identification correct rate might be due to the difficulty of obtaining satisfactory spectra from some species, such as *Mogibacterium timidum* or *Actinomyces georgiae*, and partly due to the limit of uncommon anaerobes species spectra in commercial reference libraries. Therefore, it is increasingly important to update the library of various anaerobic species, especially those lacking or poorly represented in the current version. Fortunately, commercial databases are constantly being improved and updated at intervals of about three to 6 months [48].

In this study, we analyzed two commonly used commercial MALDI-TOFMS systemstwo identification systems: the Bruker MALDI biotyper and the bioMérieux VITEK MS. To compare the same anaerobic genus between the two systems, we focused our attention on analysis of 12 out of 28 anaerobic bacteria genera included in both systems. Among them, *Bacteroides* spp., *Clostridium* spp., *Propionibacterium* spp. and *Prevotella* spp. were the predominant anaerobes. Figures 2 and 3 showed the overall identification rates of the specimens with two identification systems, MALDI biotyper and VITEK MS. The identification capacities of the two systems in Table 3 and forest plot (Figs. 2 and 3) was different. The overall identification rate of MALDI biotyper was higher than that of VITEK MS (Table 3), though the data in forest plot was opposite. It is supposed that low equipment cost leads to a wider range of MALDI biotyper applications. The rare anaerobic specimens identified by MALDI biotyper may account for a large proportion of the reason, for most of them were not included in the relevant database as previously described, which decreased of overall identification rate. This is consistent with the data presented in the forest plot.

In addition to the instrument, the identification correct ratio of anaerobic bacteria is also related to the system paired database. As shown in Table 4, one-third of the 28 studies displayed identification errors, most of which were correct genus and wrong species, and some of which were wrong genera. These results might attribute to the similarity protein composition of the species, which made the differentiation of the quality peak difficult, and made it difficult for MALDI-TOF MS to correctly identify the strain. The similarity of the protein structures led to the incorrect identification results in both anaerobic bacteria and other genera, such as *Streptococcus* spp. [49], *Mycobacterium* spp. [50], *Enterococcus* spp. [51] and yeast [52]. These result-related mistakes might be attributed to the similar protein compositions of the species, making the discrepancy of differentiation of the quality peak difficult, and making it difficult for MALDI-TOF MS to correctly identify the strains. Beyond this, the lower identification scores might be related to interspecies correlation and bacterial cell wall composition [6]. Therefore, updating the existing information and perfecting the database of difficultly identified organisms (such as *Fusobacterium* spp. and *Porphyromonas* spp.) are useful to improve the identification accuracy of MALDI-TOF MS.

Another point that cannot be ignored is the impact of the geographical distribution on the identification rate of MALDI-TOF MS. The anaerobic identification rate was slightly lower in European countries than that elsewhere. In our study, we give priority to the following reasons for this situation. The geographical distribution of the collected literature was related to the level of the economic development, given the high cost of the equipment. For this reason, the strains involved in this study reflected the situation in some developed countries rather the whole world; for instance, the technology is more advanced in Europe than that in other continents, where MALDI-TOF MS has been first applied to the field of microbial identification. In addition, MALDI-TOF MS was used to identify microorganisms with imperfect databases in some of the early studies, which is also the reason for the relatively low overall identification rate. Therefore, updating and perfecting the databases are essential for improving the identification rate. On the other hand, technological improvements should be made to reduce the cost of MALDI-TOF MS equipment as much as possible so that it can be applied in more countries and cities.

There are some limitations in our research. First, Table 3 does not list all anaerobes collected because some data of the species were discarded for statistical reasons. In additional, some articles only reported the results of MALDI-TOF MS identification of the isolates at the “species” level without providing the identification results at the “genus” level. If a specimen was not identified to “species”, it would be defined as being identified neither at the species level nor at the genus level. This may lead to the negligence of the strains identified at the “genus” level, thus underestimating the accuracy of MALDI-TOF MS in identifying anaerobes at the genus level. Despite these problems, MALDI-TOF MS is still widely used in bacterial identification and other fields such as strain typing [53], detection of virulence factors [54] and evaluation of drug resistance [55–57].

## Conclusions

In conclusion, the current meta-analysis showed that MALDI-TOF MS has shown a high degree of accuracy in anaerobic identification, although there is a lack of data to define its effectiveness in identifying rare anaerobic species. As a novel technology, MALDI-TOF MS has been widely used in the clinical diagnosis of pathogenic diseases. Therefore, it is necessary to analyze the comprehensive ability of this technique in clinical and microbiology diagnosis in the future.

## Supplementary information

**Additional file 1.** The details of 6685 strains.

## Data Availability

All data generated or analyzed during this study are included in this published article and its ***supplementary information files.***

## Abbreviations

CI: Confidence interval
MALDI-TOF MS: Matrix-assisted laser desorption ionization-time of flight mass spectrometry
